# *Trypanosoma brucei*: Trypanosome-specific endoplasmic reticulum proteins involved in variant surface glycoprotein expression

**DOI:** 10.1016/j.exppara.2010.01.015

**Published:** 2010-07

**Authors:** Ya-Nan Wang, Ming Wang, Mark C. Field

**Affiliations:** a Department of Pathology, University of Cambridge, Tennis Court Road, Cambridge CB2 1QP, UK; b College of Veterinary Medicine, China Agricultural University, Beijing 100193, China

**Keywords:** *Trypanosoma brucei*, Intracellular transport, Trypanosome, Variant surface glycoprotein, Antigenic variation, Exocytosis

## Abstract

In *Trypanosoma brucei* the GPI-anchored variant surface glycoprotein (VSG) represents ∼90% of cell surface protein and a major proportion of endoplasmic reticulum (ER) biosynthetic output. We identified four trypanosomatid-specific genes encoding candidate ER-resident proteins; all were required for normal proliferation. For Tb11.01.2640 and Tb11.01.8120, an increase in VSG abundance was found on silencing, while the protein products localized to the ER; we designated these ERAP32 and ERAP18 for ER-associated protein of 32 kDa and 18 kDa. Silencing ERAP32 or ERAP18 did not alter expression levels of ISG65 or ISG75, the major surface *trans-*membrane domain proteins. Surface biotinylation or immunoflorescence did not identify intracellular VSG accumulation, while FACS and fluorescence microscopy indicated that the cells were not increased in size, arguing for increased VSG density on the cell surface. Therefore, ERAP32 and ERAP18 are trypanosome-specific ER-localized proteins with a major role in VSG protein export and, contrary to current paradigms, VSG is not saturated on the cell surface.

## 1 Introduction

The African trypanosome, *Trypanosoma brucei*, is the etiologic agent of sleeping sickness in humans and nagana in livestock in sub-Saharan Africa and transmitted between mammalian hosts by the tsetse fly vector (Checchi and Barrett, 2008). Despite the threat to 50 million people and economic impact, the current strategy against trypanosomiasis is principally chemotherapeutic; many of these agents are toxic, limited in efficacy to specific stages of infection while drug resistance is becoming common (Fevre et al., 2008). Vaccination prospects against trypanosomiasis are effectively nullified by antigenic variation and additional immune evasion mechanisms (Field et al., 2009). In mammalian infective stages the *T*. *brucei* surface is dominated by 5 × 10^6^ homodimers of the glycosylphosphatidylinositol (GPI)-anchored variant surface glycoprotein (VSG), which form a dense coat and determine the antigenic phenotype of the cell (Morrison et al., 2009). The parasite periodically switches expression of distinct VSG variants and such switches pre-empt the immune response, avoiding destruction of the parasite at the population level by antibody-dependent killing mechanisms. In addition to VSG there is also a family of invariant surface glycoproteins (ISGs) expressed at the cell surface, albeit at substantially lower abundance of ∼10^4^ copies (Overath et al., 1994). These molecules do not exhibit antigenic variation, are anchored by a *trans-*membrane domain and have a considerably shorter half-life than VSG (Chung et al., 2008). Several families are known, of which the ISG65 and ISG75, of ∼65 kDa and ∼75 kDa apparent molecular weight, respectively, are the better characterized (Ziegelbauer and Overath 1992; Chung et al., 2004; Leung et al., unpublished data).

Importantly, the density of VSG at the cell surface is thought to be so high as to provide an essentially impenetrable barrier to the immune system, preventing recognition of other surface determinants, including the ISGs. In support of this model are observations that the most variable regions of VSG molecules are mainly restricted to the N-terminally disposed surface loops, although additional determinants have also been proposed and shown to contribute to the immune response (Field and Boothroyd 1996; Dagenais et al., 2009). Theoretical calculations based on the trypanosome surface area, crystallographic structural data and *N*-glycan modeling suggests a comparatively tight array of VSG dimers (Mehlert et al. 2002). Further, earlier studies suggested that ISG epitopes were not accessible to antibody on living cells, while in fixed cells ISGs could be recognized (Ziegelbauer and Overath, 1993).

This view of the surface is however, essentially static, and potentially inconsistent with some recent observations. Firstly, the rapidity of VSG capping suggests a highly mobile cell surface, consistent with the majority of surface protein being GPI-anchored and hence not tethered to cytoskeletal elements or tethering factors. The absence of evidence for an obvious interaction between the subpellicular microtubule array and surface proteins is also consistent with rapid uptake of both VSG and ISGs (Engstler et al., 2007; Chung et al., 2004, 2008), as well as the fast redistribution of recycled VSG across the parasite surface (Engstler et al., 2004). Recent data also suggests that ISG65 may be recognized in live cells at the cell surface, and moreover that a considerable endosomal pool was overlooked in earlier studies (Chung et al., 2004). These data all argue for a more accessible and dynamic surface than had been suggested. Finally, the molecular weights of ISG65 and ISG75, which are greater than VSG (∼58 kDa), are also difficult to reconcile with a fully occluded invariant surface protein repertoire.

In all eukaryotes newly synthesized secretory, *trans*-membrane domain and GPI-anchored proteins enter the ER to achieve their native conformation before being transported to later compartments. The ER maintains a quality control mechanism for monitoring folding of nascent polypeptides (reviewed in Brodsky and McCracken, 1999; Ellgaard and Helenius, 2003; Hebert and Molinari, 2007). Ultimately this system consists of several pathways, including hsp/dnaj chaperones, protein disulfide isomerases, the calreticulin system and also a series of lectins and glycosyltransferases; these factors collaborate to assist in attaining the native fold, in correct coupling of disulfide bonds and in monitoring the progress of the system. A timing mechanism based on hydrolysis of oligomannosyl *N*-glycans present on ER glycoproteins serves as a signal for rejection. Terminally misfolded proteins are disposed of via ER-associated degradation (ERAD), which delivers these aberrant products for degradation via dislocation into the cytosol and the ubiquitin/proteasome pathway (Kostova and Wolf, 2003; Ismail and Ng 2006; Feldman and van der Goot, 2009).

VSG biosynthesis is also initiated at the ER, where the nascent VSG is processed by GPI-addition and *N*-glycosylation and subsequently transported to the Golgi complex (Bangs et al., 1986; Vidugiriene and Menon 1994). Exocytosis to the cell surface is via the flagellar pocket (Grunfelder et al., 2003; Sutterwala et al., 2007). VSG transport from the ER to the cell surface is rapid and efficient (Bangs et al., 1986). Several small GTPases are implicated in post-ER transport of VSG to the cell surface, specifically TbRab1, TbRab2, TbRab11 and the ADP-ribosylation factor 1 (ARF1) (Dhir et al., 2004; Hall et al., 2005; Price et al., 2007). However, few molecular players in the early stages of VSG biosynthesis have been described. There is clear evidence for a role for the major ER chaperone TbBiP, and more recently an extensive RNAi screen of trypanosome ER resident factors, encompassing chaperones, calreticulin system components, protein disulfide isomerases and several other factors, suggested a considerable number of gene products are required during early biosynthesis and folding of VSG (Field et al., 2010). Moreover, a considerable biosynthetic excess is detectable when ER-associated degradation (ERAD) and/or quality control (ERQC) pathways are inhibited, either by RNAi knockdown or chemical inhibition of the proteasome with MG132 (Field et al., 2010). However, these studies did not identify truly novel trypanosome-specific factors that may have evolved specifically for VSG biosynthesis and folding.

Here we aimed to identify trypanosome-restricted ER-resident factors, and to establish if they play a role in VSG biosynthesis. We identified four potential trypanosomatid-restricted novel ER-resident proteins in the *T*. *brucei* genome, and provide evidence for a role for two of them in VSG biosynthesis. Surprisingly, for one of these gene products we were able to obtain evidence that VSG density is increased on the cell surface following knockdown.

## 2 Materials and methods

### 2.1 In silico screening for novel ER factors in the *T. brucei* genome

Initial database searches were performed on the *T*. *brucei* genome at geneDB (http://www.genedb.org/) using the following search criteria: (i) predicted N-terminal signal peptide, (ii) C-terminal degenerative [K/H]DEL sequence, (iii) lack of annotation as a clear orthologue to a higher eukaryote gene, and (iv) absence of a clear annotated domain, either at geneDB or subsequently at pfam (http://pfam.sanger.ac.uk/). Signal peptides were predicted with the SignalP 3.0 program (Bendtsen et al., 2004). The blastp program (http://www.ncbi.nlm.nih.gov/BLAST/) was used to search additional genome databases. Multiple protein sequence alignments were carried out using ClustalW (http://www.ebi.ac.uk/clustalw/), and paired alignment was performed with T-coffee (http://tcoffee.vital-it.ch/cgi-bin/Tcoffee/tcoffee_cgi/index.cgi). Alignment results were visualized using ESPript (http://espript.ibcp.fr/ESPript/ESPript/index.php).

### 2.2 Trypanosomes and cell culture

Bloodstream cells of *T*. *brucei* Lister 427 (wild-type 427, WT427) and the single marker bloodstream (SMB) (Wirtz et al., 1999) were cultured in HMI-9 complete medium (Gibco) (Hirumi and Hirumi, 1989) supplemented with 10% heat-inactivated fetal bovine serum (FBS) (Biosera), penicillin/streptomycin (Gibco) and l-glutamine (Gibco), maintained at 37 °C with 5% CO_2_ in a humid atmosphere as described previously (Leung et al., 2008). For tetracycline-inducible SMB-derived lines, neomycin (G418, Sigma) and hygromycin B (Invitrogen) were supplemented in the medium at final concentration of 2.5 μg/ml. Procyclic form *T*. *brucei* cells were maintained in SDM-79 (Gibco) medium at 27 °C, supplemented with 10% FBS, penicillin/streptomycin (Gibco) and l-glutamine (Gibco).

### 2.3 Recombinant DNA constructs

Primers for amplification of RNAi target fragments were designed using RNAit (Redmond et al., 2003). Forward (F) and reverse (R) primers (all sequences are written 5′ to 3′), respectively, were GGTTGTGTTCAGGCTTGGTT and TAAAATACGGGAAATGCCCA for Tb11.01.2640 (ERAP32), AACCCAAAACACGAGGAGTG and TTCTGCTTTGTTCTCCGCTT for Tb927.7.3870, AGCTCAGAGTGCCCTTATCG and TTACCCCATGACTGATTCCG for Tb927.2.5140, and CTTCATGGCTGTCCTTCGAG and CGCATCTTTTACCCCAAGAA for Tb11.01.8120 (ERAP18). All PCR products were amplified from *T*. *brucei* genomic DNA using Taq DNA polymerase (Sigma) and cloned into p2T7^TA^ (LaCount and Donelson, 2001) to generate the corresponding RNAi plasmids: p2T7^TA^-ERAP32, p2T7^TA^-Tb927.7.3870, p2T7^TA^-Tb927.2.5140, and p2T7^TA^-ERAP18. For creation of haemagglutinin (HA) tag fusion constructs, two more sets of primers were generated for ERAP32 (F: cgtAAGCTTATGGCGTCCTGCGTGAC and R: cgtGAATTCTCACAACTCTTTTTCCGCGTAGTCTGGAACGTAGGGGTATCGAACTAAAATAC) and ERAP18 (F: acgAAGCTTATGAGTTCTTCATGGCTG and R: cgtGAATTCTCATAGCTGCTCATCCGCGTAGTCTGGAACGTCGTAGGGGTATGTCGCATCTTTTAC). Restriction sites for cloning purposes are shown in italic. The HA-tag sequence was inserted into the open reading frame (ORF) of individual candidate before the ER-extension motif sequence to create the C-terminal HA-fusion protein. The corresponding construct was cloned into expression vector pXS5 (Chung et al., 2008) by using HindIII and EcoRⅠ sites. The resultant plasmids, pXS5-ERAP32 and pXS5-ERAP18 were transfected into BSF WT427 parasites. Clonal transformants were selected by resistance to 2.5 μg/ml G418 (Sigma). For transfection pXS5 and pXS2 vectors were linearized by XhoI or NotI, respectively.

### 2.4 Transfection of BSF *T. brucei*

Transfections were performed using the Amaxa human T-Cell Nucleofector® kit (Amaxa, Koeln, Germany) following the manufacturer’s guidelines with some modification. Briefly, cells (3 × 10^7^ log phase bloodstream cells) were harvested at 800 g for 10 min at 4 °C, then suspended in 100 μl of ice-cold Amaxa Human T-Cell solution. Following the addition of 10 μg of linearized DNA plasmid the sample was transferred to a cuvette and transfected with the Amaxa Human Nucleofector® II contraption, program X-001. Electroporation mixtures were immediately transferred to flasks containing pre-warmed HMI-9 complete medium. After 6 h, selection antibiotic was added to each flask and distributed into a 24-well plate and then incubated at 37 °C as described above. Positive transformants were selected on the 5th or 6th day after transfection. Cells without linearized DNA plasmids were used as negative electroporation controls, which routinely exhibited no antibiotic resistant cells.

RNA interference and proliferation analysis: Linearized plasmids (p2T7^TA^-ERAP32, p2T7^TA^-Tb927.7.3870, p2T7^TA^-Tb927.2.5140, and p2T7^TA^-ERAP18) were transfected into SMB cells as described above. Positive clones were selected by culturing cells in the constant presence of 2.5 μg/ml G418 and hygromycin B. Induction of RNAi was initiated by adding tetracycline at a final concentration of 1 μg/ml to the culture. Triplicate cultures were initiated at 5 × 10^4^ cells/ml with or without tetracycline for monitoring proliferation continually for 5 days. Every 24 h, cell density was determined by counting trypanosomes with a Z2 Coulter Counter (Beckman), the culture diluted back to 5 × 10^4^ cells/ml, counted again and returned to the incubator.

Quantitative real-time polymerase chain reaction (qRT-PCR): Total RNA was extracted using the RNeasy mini kit (Qiagen) from 1 × 10^8^ BSF cells according to the manufacturer’s instructions. The concentration of RNA was measured twice by a ND-1000 spectrophotometer and Nanodrop software (Nanodrop Technologies) before transcription. 2 μg of total RNA from each sample was reverse transcribed using a SuperScript® II Reverse Transcriptase kit (Invitrogen). qRT-PCR was performed by using iQ-SYBRGreen Supermix and the MiniOpticon Real-Time PCR Detection System (Bio-Rad). Forward and reverse primers were used as followed: GAAAACGAAGGGATGCGTTA and AGGAGATGAGCAGCGTGTTT for ERAP32, TCAAAGCTATGTCACCACAGG and TTTCGCTCTGCCGTAAAAAT for Tb927.7.3870, and AACGCGCTAAGGATGAGGAT and CTTGATCTTCATTACCCCATGA Tb927.2.5140, and GCTGTGCCTATTGAGGAGGTT and TGCTCATCTGTCGCATCTTT for ERAP18. The qRT-PCR reaction was performed using a MiniOpticon (Bio-Rad) as previously described (Koumandou et al., 2008), using β-tubulin as an internal standard.

### 2.5 Western blot analysis

Parasites were harvested at 800 g for 10 min at 4 °C after 24 h induction and washed once with ice-cold phosphate-buffered saline (PBS) (tablets from Sigma). Samples were resuspended in 50 μl PBS and mixed with 50 μl 2xsodium dodecyl sulfate–polyacrylamide gel electrophoresis (SDS–PAGE) sample loading buffer. Samples were heated to 95 °C for 15 min and then subjected to SDS–PAGE, electroblotted onto Immobilon-P membrane (Millipore Corp.) and probed with appropriate antibodies. Rabbit anti-BiP (gift from J. Bangs, Madison, WI, USA), rabbit anti-VSG221, rabbit anti-ISG65, rabbit anti-ISG75 (gifts from M. Carrington, Cambridge, UK), and anti-HA epitope immunoglobulin G (IgG) (Santa Cruz Biotechnology) were applied as primary antibody for 1 h at room temperature at 1:10,000, 1:5000 and 1:5000, 1:5000, and 1:10,000 dilution in blocking buffer (1% skimmed milk in TBST (137 mM NaCl, 2.7 mM KCl, 25 mM Tris base, pH 7.4, 0.2% Tween 20)), respectively. After three washes with TBST, anti-rabbit or anti-mouse peroxidase-conjugated secondary antibody (both from Sigma) was applied at 1:10,000 dilution and incubated for 40 min at room temperature. Bound-antibodies were detected by reaction with chemiluminescence using Biomax MR-1 films (Kodak). Films were scanned and quantitated using ImageJ software (NIH).

### 2.6 Immunofluorescence analysis

For immunofluorescence analysis, cells were harvested by centrifugation, and resuspended in ice-cold Voorheis’s-modified phosphate-buffered saline (vPBS; PBS supplemented with 10 mM glucose and 46 mM sucrose, pH 7.6). After fixation, by mixing with an equal volume of 6% paraformaldehyde and incubating on ice for 10 min, cells were spread onto poly-l-lysine microscope slides (VWR International) and allowed to adhere at room temperature for 30 min. If appropriate, permeabilization was performed by incubating with 0.1% Triton X-100 in PBS for 10 min at room temperature followed by three washes in PBS. For analysis of RNAi cell lines, 30 × 10^7^ cells of induced or uninduced cells 24 h post-induction were harvested by centrifugation. The following primary antibodies were used: anti-BiP (1:1000), anti-VSG221 (1:1000), anti-ISG65 (1:1000), and anti-ISG75 (1:1000) in 10% FBS solution. Oregon Green 488 or Alexa Fluor 488 secondary antibodies (Sigma) were applied, both at 1:1000 dilution. After three washes in PBS, slides were dried and then mounted with 4′,6-diamidino-2-phenylindole (DAPI) (Vector Laboratories, Inc.) and sealed with nail varnish (Max Factor Inc.). For analysis of localization of HA-tagged proteins, procedures were performed as described above with the exception that mouse anti-HA as primary antibody (1:1000) and Oregon Green 488 as secondary antibody (1:1000) were used.

Surface biotinylation: 1 × 10^7^ cells were washed three times with ice-cold vPBS and then exposed to 1 mM EZ-Link™ sulfosuccinimidyl-6-(biotinamido) hexanoate (Sulfo-NHS-LC-Biotin) (Pierce) by rolling for 1 h at 4 °C in washing buffer (130 mM NaCl, 5 mM KCl, 1 mM CaCl_2_,1 mM MgSO_4_, 5 mM NaPO_4_, 20 mM HEPES, pH7.1). After a further three washes, cells were lysed by incubating with lysis buffer (150 mM NaCl, 1% Triton X-100, 0.1% SDS, and 20 mM Tris buffer, pH7.4), and rolling for 1 h at 4 °C in the presence of protease inhibitors (Roche). Biotinylated proteins were then bound to EZview Red Streptavidin Affinity Gel (Sigma) by gently shaking for 1.5 h at 4 °C. The beads were washed three times with wash buffer and adsorbed proteins were eluted with 50 μl 2 × SDS–PAGE loading sample buffer by boiling for 5 min. Non-biotinylated proteins were precipitated by 10% trichloroacetic acid (TCA) buffer and resolved in 50 μl 2 × SDS–PAGE loading sample buffer. Proteins were loaded onto SDS–PAGE gels, electroblotted to Immobilon-P membrane and probed with anti-VSG and anti-BiP antibodies. Band intensities were quantified by densitometric analysis using ImageJ software.

### 2.7 VSG export

The assay was performed as described (Allen et al., 2003). Briefly, mid-log phase BSF cells were incubated with labeling medium (Met/Cys-free Dulbecco’s modified Eagle’s medium (Sigma)) supplemented with 10% dialyzed FBS at 37 °C for 30 min. Next, cells were pulse-labeled at 37 °C for 7 min with EasyTag™ EXPRESS^35^S Protein Labeling Mix (PerkinElmer) at a specific activity of 200 μCi/ml, then diluted 1:10 with pre-warmed complete HMI-9 medium, and chased for up to 1 h at 37 °C. During the chase period, aliquots were removed at 0, 5, 10, 20, 30, 45 and 60 min post-chase and placed on ice. Cells were incubated at 37 °C for 10 min to enable the GPI-specific phospholipase C to convert susceptible membrane-form VSG (mfVSG) to soluble VSG (sVSG) and then lysed in lysis buffer (150 mM NaCl, 1% NP-40, 50 mM Tris HCl, pH 7.5, and protease inhibitor). The two different forms of VSG were separated by centrifugation for 10 min at 20,000*g* at 4 °C. Labeled VSG was recovered by incubation for 1 h at 4 °C with ConA Sepharose 4B (Sigma) in the presence of 1 mM CaCl_2_, 1 mM MnCl_2_ and ConA wash buffer (150 mM NaCl, 1 mM CaCl_2_, 1 mM MnCl_2,_ and 10 mM Tris HCl, pH 7.5). Finally, samples were resuspended in 2 × SDS–PAGE loading buffer and loaded onto SDS–PAGE gels at 1 × 10^6^ cells/lane. Gels were stained, fixed, dried and radiolabeled proteins were detected by autoradiography.

### 2.8 Fluorescence activated cell sorting (FACS) analysis

Mid-logarithmic phase growth cells were harvested at 800 g for 10 min at 4 °C and washed once in cold PBS. For cell size analysis, 1 × 10^6^ cells were resuspended in 0.5 ml cold PBS with 3 μM Hoechst 33342 final concentration (Sigma), and incubated at 37 °C for 30 min. The suspension was mixed with 0.5 ml 2% formaldehyde in PBS and measured thereafter using a 11 parameter Cyan ADP (Beckman Coulter) at 30 mW UV. Single cells were gated away from clumped and dying cells by using Pulse-width versus UV-2 plots. 1N and 2N cells were gated from UV-2 versus counts plots. Relative cell sizes were displayed in a forward scatter (FSC) liner versus counts plots. For surface VSG detection, 1 × 10^6^ cells were suspended in 0.5 ml cold PBS and then fixed with 0.5 ml 2% formaldehyde in PBS for 30 min. After fixation, cells were incubated with rabbit anti-VSG221 (1:200 in 10% FBS) at room temperature for 30 min and then washed once in PBS. Alexa Fluor 488 was used as secondary antibody at 1:200 dilution in 10% FBS. Cells were stained with Alexa Fluor 488 for 30 min at room temperature and intensities of VSG were measured afterwards using a Cyan ADP at a power setting of 30 mW FL2. Clumped cells were gated away in FL-2 log versus FSC plot. The surface VSG intensities were measured using FL-2 log versus counts plot. For each event, 30,000–50,000 cells were counted, and data were processed with Summit 4.3 software (Beckman Coulter).

### 2.9 Electron microscopy analysis

1 × 10^8^ uninduced and induced cells from ERAP32 and ERAP18 RNAi cell lines were harvested and washed once in saline (0.98% NaCl in 100 mM HEPES pH 7.0). Cells were subsequently fixed in 4% (v/v) glutaraldehyde for 2 h, washed in 0.1 M HEPES buffer three times, post-fixed with 1% Osmium tetroxide for 1 h, washed once with distilled water, bulk-stained in uranyl acetate for 1 h, dehydrated with a series of ethanol solutions, and embedded. Sections were cut at 0.7 μm on a Leica Ultracut E microtome, and stained with 1% methylene blue for 2 min, then, viewed in a Philips CM100 (FEI-Philips).

## 3 Results

### 3.1 In silico identification of candidate novel ER-targeted proteins

**Fig. 1:**
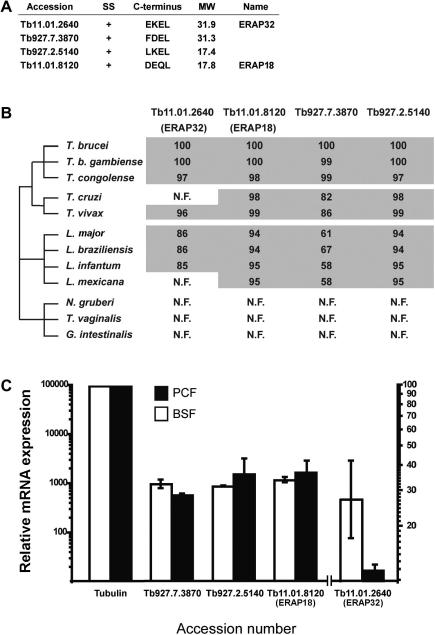
Identification and distribution of candidate novel ER proteins in *Trypanosoma brucei*. (A) GeneDB accession numbers and properties of the four candidate ER proteins selected for study. SS, signal peptide; C-terminus, C-terminal ER-extension like sequence; MW, molecular weight in kDa; +, N-terminal signal peptide predicted; Name, proposed name. (B) Distribution of candidate proteins across selected Excavata genomes. N.F. represents that evidence for an ortholog could not be found in the relevant taxon. Numbers represent the percent similarity by pairwise alignment with the *T. brucei* sequence as reference, using T-coffee. (C) mRNA expression levels of candidate ER proteins in PCF (black bars) and BSF (white bars) cells by qRT-PCR. Expression was normalized to tubulin. The experiment has been done a total of three times on different mRNA samples. Bars indicate the standard error.

Multiple potential ER-targeted or ER-resident proteins can be identified in the *T*. *brucei* genome. Four of these are annotated as hypothetical proteins and are the subject of this paper. All four hypothetical proteins contain predicted N-terminal signal peptides and degenerate C-terminal ER-retention-like motifs (Fig. 1A). Two of them, Tb927.2.5140 and Tb11.01.8120 are quite small, with predicted molecular weights of 17.4 and 17.8 kDa, respectively, while Tb11.01.2640 and Tb927.7.3870 have molecular masses of ∼31.9 and −31.3 kDa, respectively. Tb927.7.3870 contains a predicted thioredoxin-like domain, with a low score for sequence similarity of the conserved domain. In addition one or two *trans*-membrane domain helices are predicted, but more detailed examination of these using the Kyte–Doolittle algorithm revealed that these are all comparatively weak predictions, and only Tb11.01.2640 is likely membrane-anchored at the N-terminus, leaving the C-terminal EKEL probably in the ER lumen (data not shown).

Investigation of the evolutionary distribution of these genes across the Excavata revealed orthologues in most trypanosomatids; all but Tb11.01.2640 were universally represented in nine Kinetoplastida genomes (Fig. 1B). The sequence of Tb927.2.5140 and Tb11.01.8120 are extremely well conserved, exhibiting ∼90% amino acid similarity between orthologues, while Tb927.7.3870 orthologues are almost completely conserved between African trypanosome subspecies, but less so in *Trypanosoma* *cruzi*, *Trypanosoma* *vivax* and Leishmania species. Tb11.01.2640 was not found in *T*. *cruzi* and *Leishmania mexicana*, but due to representation in other Leishmania species, this is likely the result of secondary losses.

**Supplementary Fig. 1:**
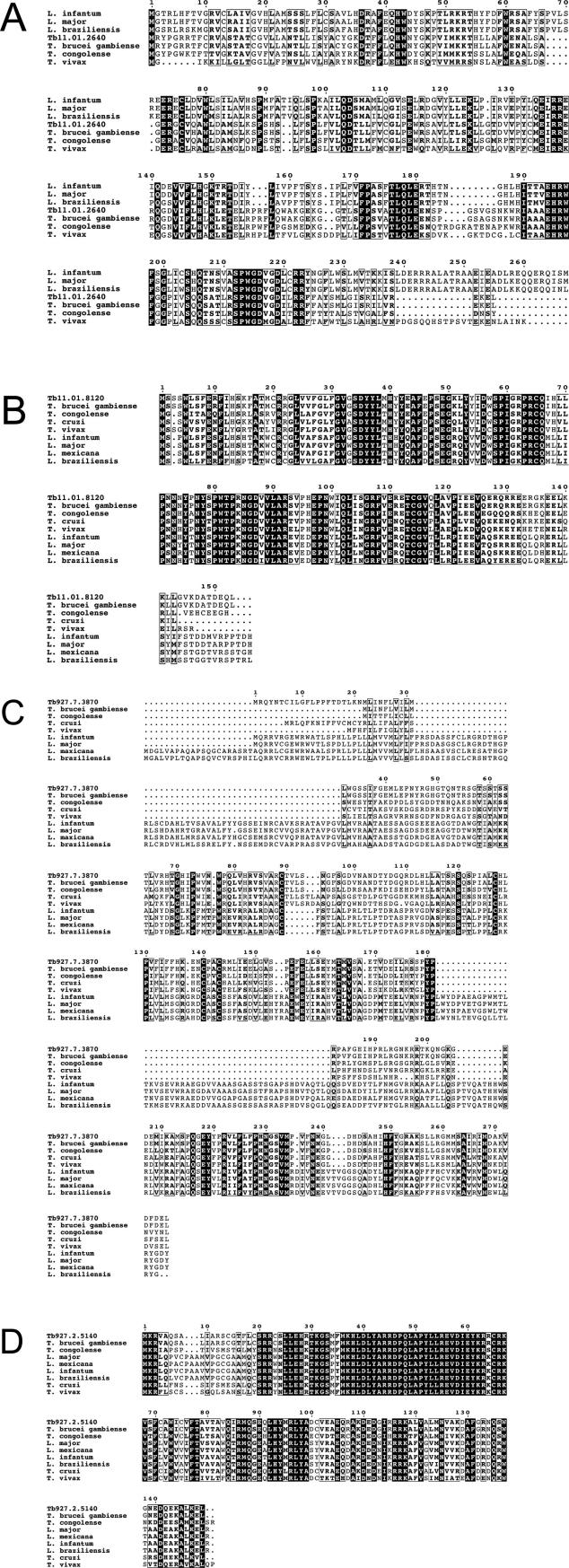
Multiple sequence alignments of predicted amino acid sequences of four candidate ER proteins against their orthologues from selected Kinetoplastida genomes. Each homologue for each candidate was given the name based on the corresponding species name. Identical and similar amino acids are highlighted in black or outlined, respectively. A. Alignment for ERAP32 (Tb11.01.2640) orthologues across seven species. Accession numbers in vertical order: LinJ36_V3.5280, LmjF36.5050, LbrM35_V2.5300, Tb11.01.2640, gamb1662a11_1859d04, TcIL3000.11.11540, and Tviv1152c05.p1k_1. B. Alignment for ERAP18 (Tb11.01.8120) orthologues across nine species. Accession numbers in vertical order: Tb11.01.8120, Tbgamb.42801, TcIL3000.11.16350, Tc00.1047053506727.150, TvY486_1117370, LinJ32_V3.3890, LmjF32.3740, LmxM31.3740, LbrM32_V2.3980. C. Alignment of Tb927.7.3870 orthologues across nine species. Accession numbers in the vertical order: Tb927.7.3870, Tbgamb.14951, cIL3000.7.3130,Tc00.1047053511249.80, TvY486_0703860, LinJ14_V3.1150, LmjF14.1080, LmxM14.1080, LbrM14_V2.1070. D. Alignment of Tb927.2.5140 orthologues nine species. Accession numbers in vertical order: Tb927.2.5140, Tbgamb.2156, TcIL3000.2.1250, LmjF27.2370, LmxM27.2370, LinJ27_V3.2320, LbrM27_V2.2580, Tc00.1047053511627.80, tviv623d01.q1k_21.

Evidence for sequences related to the four genes could not be found by searching three complete non-trypanosomatid Excavata genomes, specifically *Naegleria* *gruberi*, *Trichomonas vaginalis* and *Giardia intestinalis* (Fig. 1B), nor in non-excavate taxa (data not shown), suggesting that these genes arose at the time of origin of the *Trypanosoma* lineage and hence may play a unique biological role in trypanosomes. The high level of sequence conservation of these four genes supports this hypothesis (Fig. 1B and Supplementary Fig. 1).

Expression of all four genes in both bloodstream (BSF) and procyclic (PCF) trypanosomes was demonstrated by qRT-PCR (Fig. 1C). With the exception of Tb11.01.2640, the mRNA levels are reasonably abundant, at ∼1.0% of tubulin levels. By contrast, the mRNA level of Tb11.01.2640 was ∼10^5^-fold less abundant than tubulin in both life stages examined, and was also the only gene exhibiting significant developmental regulation, being more abundant in the BSF stage. Tb11.01.2640 was also found to be low abundance by microarray analysis (Koumandou et al., 2008).

### 3.2 Down-regulation of all four candidate ER gene products affects cell proliferation

**Fig. 2:**
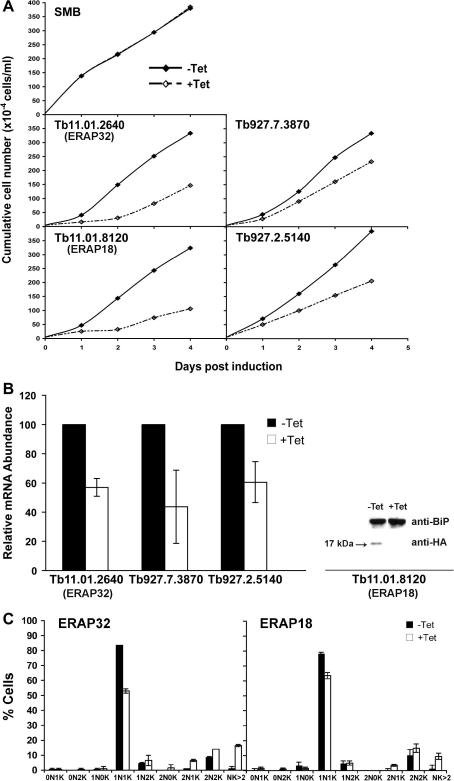
Effect of RNAi against candidate genes on proliferation of BSF *T. brucei*. (A) Cumulative proliferation curves for each of four candidates. RNAi cell lines were cultured in the presence (+Tet) or absence (−Tet) of 1 μg/ml tetracycline. The experiment for each candidate was carried out in triplicate with essentially identical results, and included the SMB cell line as a negative control. (B) Validation of targeting specificity. The mRNA levels of three genes, Tb11.01.2640 (ERAP32), Tb927.7.3870 and Tb927.2.5140 was measured by qRT-PCR and normalized to tubulin. Results are presented as the mean ± SD from two independent experiments. Down-regulation of Tb11.01.8120 (ERAP18) was addressed by Western blotting against an HA-tagged version of the gene product expressed from an integrated pXS5 plasmid. Lysates were probed with anti-HA and also anti-BiP as loading control. (C) Numbers of nuclei (N) and kinetoplasts (K) in ERAP32 and ERAP18 RNAi cell lines. DNA was visualized by DAPI staining followed by examination under a fluorescence microscope. Error bars represent mean ± SD from analysis of 300 cells in three independent experiments.

To investigate the functions of the four genes, initially the effects of RNAi knockdown, using the tetracycline-inducible p2T7^TA^ vector in *T. brucei* single marker bloodstream (SMB) cells, were examined. Proliferation defects were observed following 1 day of RNAi induction in each induced RNAi cell line. Proliferation defects caused by silencing Tb11.01.2640 and Tb11.01.8120 were very pronounced (Fig. 2A, lower left panels), while the growth impact was substantially less prominent for the remaining two genes (Fig. 2A, lower right panels).

Down-regulation at the mRNA level of three genes could be confirmed by qRT-PCR after 1 day’s induction (Fig. 2B, left panel). These data indicated that mRNA levels in the Tb11.01.2640, Tb927.7.3870 and Tb927.2.5140 induced RNAi cell lines were reduced by around 43%, 56%, and 49% compared to the corresponding uninduced cell lines. This comparably modest impact on mRNA levels is likely in part due to the time of analysis, selected as the earliest point where a clear proliferation defect was manefest and therefore minimizing potential non-specific suppression due to pleotrophic effects. It was not possible to detect a PCR-amplified product by qRT-PCR for Tb11.01.8120 as the RNAi construct p2T7^TA^-Tb11.01.8120 essentially contained the entire ORF. However, depletion of HA-tagged Tb11.01.8120 protein in SMB cells lines containing both an HA-Tb11.01.8120 transgene and the p2T7 ^TA^-Tb11.01.8120 RNAi construct was possible by Western blotting of whole cell lysates collected from induced cells 1 day post-induction (Fig. 2B, right panel).

Knockdown of Tb11.01.2640 and Tb11.01.8120 also led to accumulation of cells with abnormal morphologies, suggesting that these gene products may play some role in cell division. In induced cells, abnormal numbers of nuclei and kinetoplasts appeared, including 1K2N, >2K2N, accompanied by a decreased number of cells with 1K1N (Fig. 2C). This was rather more pronounced for Tb11.01.2640 than Tb11.01.8120, but overall the effect was comparatively mild, with the vast majority of cells in the population retaining normal organellar numbers, i.e. 1K1N, 2K1N or 2K2N. While these data suggest that Tb11.01.2640 and Tb11.01.8120 depletion leads to a mild cytokinesis block, later investigations indicated a role in ER function, and it is rather likely that these effects are secondary, and result from compromising normal ER processes.

### 3.3 Tb11.01.2640 and Tb11.01.8120 encode ER-localized proteins

**Fig. 3:**
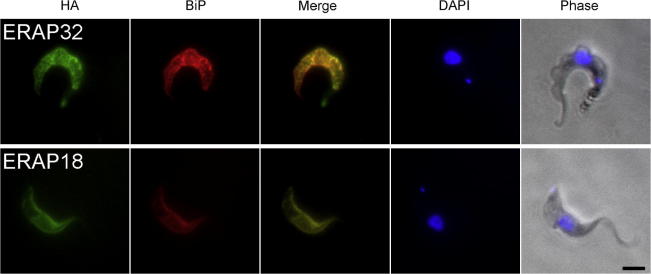
Localization of ERAP32 and ERAP18 HA-tagged proteins in BSF *T. brucei*. HA-tagged versions of ERAP32 and ERAP18 were stably expressed by integration of pXS5 plasmids harboring epitope-tagged versions of ERAP18 or 32, and the tagged protein visualized with a mouse monoclonal anti-HA antibody followed by a secondary antibody (in green). The ER was stained with rabbit polyclonal anti-TbBiP antibody and visualized with FITC-conjugated secondary antibody (red). DNA was stained with DAPI (blue). Scale bar 2 μm.

To determine which, if any, of the products of Tb11.01.2640, Tb11.01.8120, Tb927.7.3870 and Tb927.2.5140 were present in the ER we inserted a single HA9 epitope tag into each ORF N-terminal to the C-terminal ER retention motif. ORFs were then subcloned into the pXS5 expression vector and the corresponding linearized plasmids were transfected into BSF 427 trypanosomes. Positive transformants were costained with TbBiP, an ER-resident protein and ER marker, and HA (Fig. 3). Fluorescence for both Tb11.01.2640 and Tb11.01.8120 fusion proteins exhibited extensive overlay indicating that they likely encode ER-resident proteins. Despite attempts with HA or GFP-tagging at multiple sites, or by genomic tagging, no expression of tagged versions of Tb927.2.5140 or Tb927.7.3870 could be detected (data not shown), suggesting that overexpression or tagging of these gene products may be detrimental. Tb11.01.2640 and Tb11.01.8120 are therefore authentic ER-localized proteins and we designated them as ERAP32 and ERAP18, for ER-associated proteins of 32 kDa and 18 kDa, respectively.

### 3.4 Knockdown of ERAP32 and ERAP18 in BSF *T. brucei* results in increased levels of VSG

**Fig. 4:**
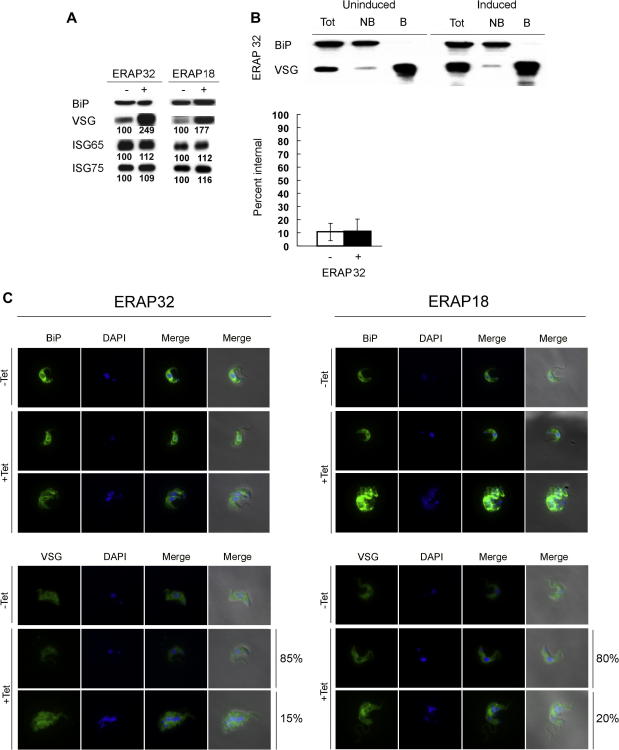
Alterations to VSG expression levels in ERAP32 and ERAP18 knockdown cells. (A) Western blot analysis of VSG abundance on RNAi induction. 1 × 10^7^ cells were sampled from either induced (+) or uninduced (−) ERAP32 and ERAP18 RNAi cell lines after 1-day induction. Equivalent number of cells (1 × 10^6^ cells) were applied to each lane. Primary antibody (rabbit anti-VSG221, rabbit anti-ISG75, rabbit anti-ISG65 or rabbit anti-BiP) was applied for 1 h at room temperature and secondary antibody was incubated with membrane for 40 min at room temperature. Pair numbers in each box indicate the relative abundance antigen in uninduced and induced cells from each RNAi cell line as determined by densitometry and normalized to the uninduced level. (B) Detection of VSG by surface biotinylation. Western blot is a representative biotinylation experiment performed on ERAP32 RNAi cell line at 12 h after induction. Tot: total VSG, NB: non-biotinylated, i.e. intracellular VSG, B: biotinylated, i.e. surface VSG. BiP was used as a loading and membrane integrity control. The lower graph represents quantification of VSG intensity from biotinylation assays following densitometry. Error bars represented mean ± standard error from at least two independent determinations. (C) IFA analysis of permeabilized cells silenced for ERAP32 and ERAP18. Upper panel: Visualization of ER structure and morphology of the cells in *T*. *brucei* stained with BiP. Lower panel: Distribution of VSG. Columns in each panel (from left to right): BiP/VSG stain (green); DAPI-stain of the nucleus and kinetoplast (blue); merged images with color combination for BiP/VSG and DAPI fluorescence; merged pictures from phase, DAPI and BiP/VSG. The first rows in each panel: uninduced cells (−Tet). The second and third rows in each panel are induced cells with normal morphology and abnormal morphology cells, respectively, and the percent representation of each morphology in the population indicated (cells with maintained normal morphology, and cells with defect in cell division and morphological abnormalities, respectively). Scale bar: 2 μm.

Since ERAP32 and ERAP18 are likely ER-resident proteins, we considered the possibility that they have a role in ER function, and hence could participate in the VSG secretory pathway. To address this possibility, we performed Western blotting on induced RNAi cell lines probing with anti-*T*. *brucei* VSG221, ISG75, ISG65 antibodies. Interestingly, depletion of ERAP32 and ERAP18, which also showed significant growth defects, evoked increased levels of VSG221 1 day post-induction (Fig. 4A), but similar changes to abundance were undetected against neither ISG65 or ISG75. Significantly we have also detected increased VSG levels under conditions that disrupt ER quality control, including inhibition of the proteasome and knockdown of multiple ER-resident chaperones and quality control factors (Field et al., 2010). Hence these data may suggest a role in ER quality control, degradation or a related mechanism for ERAP32 and ERAP18. For Tb927.2.5140 and Tb927.7.3870 there was no change in abundance of either VSG or ISG (data not shown). Considering both the failure to localize Tb927.7.3870 and Tb927.2.5140 and the absence of evidence for involvement in VSG secretory pathway so far, Tb927.2.5140 and Tb927.7.3870 were not investigated further. As the dramatic increase in VSG levels following down-regulation of ERAP32 and ERAP18 appeared by day one of induction, all the following experiments were performed with cells induced for 1 day, unless otherwise stated.

### 3.5 Additional VSG in ERAP32 and ERAP18 knockdown cells is not accumulated internally

VSG constitutes ∼10% of total protein in *T*. *brucei*, most of which is at the cell surface, contributing ∼90% of surface protein. Modeling studies have suggested that VSG at the surface is essentially saturated (Grunfelder et al. 2002; Mehlert et al. 2002), and the extreme density of VSG is even visible as an electron dense coat by transmission EM. Hence we expected that the increase in total VSG was most probably explainable as intracellular accumulation.

To determine whether this is indeed the case, we used surface biotinylation and a biotin reagent that is non-membrane permeant. Initially, we performed the experiment on cells induced for 24 h, but numerous intracellular proteins were also biotinylated in the induced cells, probably due to the severe effects that these knockdowns have on cell proliferation and viability (data not shown). Therefore, we repeated the analysis after 12 h of induction. Unfortunately, induced ERAP18 cells were extremely fragile and with clear evidence of lysis occurring as an intracellular BiP were detected by biotinylation (data not shown); therefore we restricted further analysis to ERAP32. We observed increased VSG in the cell surface protein pool of induced cells compared to uninduced cells from ERAP32 RNAi cell line, in agreement with Western blot analysis of whole cell lysates (Fig. 4B). Unexpectedly, there was little change in the ratio between intracellular VSG and surface VSG, and certainly not a high level of non-biotinylated VSG, suggesting an absence of significant intracellular VSG accumulation. When we analyzed cells by immunofluorescence neither RNAi cell line arguing against significant internal VSG accumulation, which would manifest as distinct intracellular puncta (Fig. 4C). Small puncta were observed in some cells (data not shown), but these could not account for the total increase in VSG levels, nor were they frequently observed within the induced culture population. Overall these data suggest that much of the additional VSG is surface accessible.

### 3.6 Accumulation of VSG is not due to cellular enlargement

Due to the presence of abnormal cells in the induced cultures, we wished to determine if the overall size of cells in the population had increased sufficiently to allow incorporation of the increased levels of surface exposed VSG, without a change to density at the surface. We used both microscopy to measure the length of individual cells, and also light scatter FACS analysis. Given the absence of a significant cell cycle block, we considered the possibility that cells were accumulating at a late point in the cell cycle, where they are larger, to be unlikely, but addressed this by analysis of 1K1N and/or 2K2N cells.

**Fig. 5:**
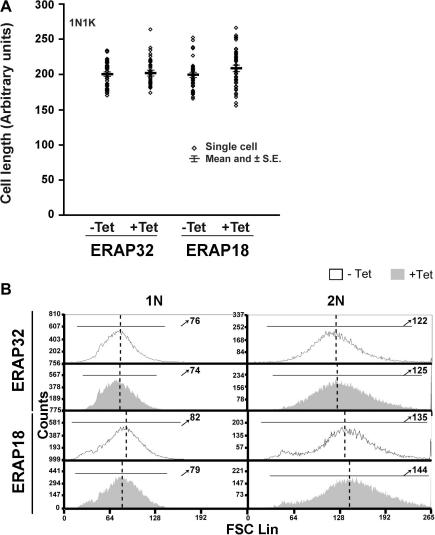
Morphometric analysis of consequences of RNAi of ERAP32 and ERAP18. (A) Cell length determined by phase contrast microscopy. Cells, either induced or uninduced with tetracycline for 1 day were stained with DAPI to visualize cell morphology. The length of cells was measured by drawing along the midline of the cell using Metamorph software. Data are presented individually and also as mean ± SD of 50 cells for each sample in a scatter plot. (B) Fluorescence histograms showing relative size of cells of either induced or uninduced cells of ERAP32 and ERAP18 from one representative FACS experiment. FSC: forward scatter. Numbers pointed by arrows represent the relative median value of cell size of corresponding group. The analysis has been done at least twice with highly similar results.

Despite apparent cell cycle defects in both RNAi cell lines, the major population remained 1N1K and 2N2K (Fig. 2C), a proportion of which exhibited atypical morphology in induced cells. By microscopy we could not measure the 2N2K cells accurately and hence restricted our analysis to 1N1K. This also served to eliminate some of the variability in cell size that is due simply to normal cell cycle progression. We were unable to detect any significant difference in the length of the 1N1K cells in either of the induced RNAi lines, when compared to uninduced control cells (Fig. 5A). To confirm this observation and to increase the number of cells analyzed, we also performed FACS analysis, monitoring forward light scattering. In this case, by gating based on nuclear DNA content, we were able to resolve 1 N (2n) and 2 N (4n) populations. The results from this analysis confirmed the microscopy analysis, i.e. there was no major change to cell size accompanying knockdown of ERAP32 or ERAP18 (Fig. 5B).

### 3.7 Increased VSG at the cell surface in knockdown cells

Given the evidence above that neither 1N1K cells nor 2N2K cells were enlarged following RNAi of ERAP32 or ERAP18, plus the biotinylation and Western blot data suggestive of increased surface VSG, we sought to confirm this finding.

**Fig. 6:**
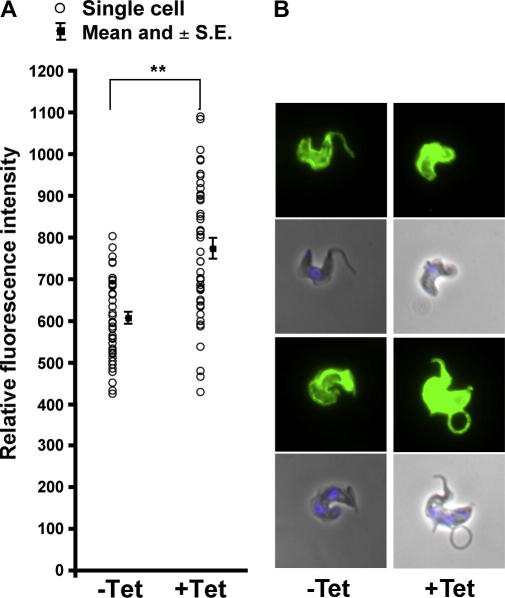
Measurement of VSG intensity on the cell surface of trypanosomes in ERAP32 RNAi cells. (A) Fluorescence intensity comparison for VSG between induced and uninduced cells without permeabilization following 1 day induction of ERAP32 RNAi. (A) VSG intensity was measured on individual cells by fluorescence microscopy. The VSG-associated fluorescent intensity of each cell was measured under identical exposure parameters. Data are presented individually and also as mean ± SD of 50 cells for each sample in a scatter graph. ∗∗*P* < 0.01. (B) Two representative images from uninduced and induced cells stained for VSG.

Firstly, we measured the fluorescence obtained by staining RNAi cells for VSG221 in the absence of permeabilization with anti-VSG221 antibody. This analysis uncovered a highly significant increase of surface VSG fluorescence (∼1.25 times, Fig. 6A and B), albeit less than that obtained by Western blotting of whole cell lysates (∼2.5 times, Fig. 4A). The attenuated increase in signal we attribute to steric effects with binding of anti-VSG antibodies to the cells, as has been demonstrated previously (Grunfelder et al., 2003). To further confirm these observations we also performed FACS to detect surface VSG with anti-VSG221 antibody in ERAP32 and ERAP18 RNAi cell lines on day one post-induction. These data also detected a portion of induced ERAP32 cells with more VSG detected at the cell surface following knockdown, resulting in a ∼1.3 times increase in signal overall (data not shown). Again, we failed to observe similar results for ERAP18 RNAi cells either by microscopy or FACS (data not shown), due to the severe fragility of these cells.

The lower levels of increased VSG detected in these surface staining assays compared to that found for cell lysates is probably due to the environment of the VSG in each case. Non-stoichiometric labeling of surface VSG, even in the presence of excess anti-VSG antibody, has been noted previously, and is presumed to result from molecular crowding preventing antibody from binding all VSG molecules (Grunfelder et al., 2003). Given that the evidence here suggests increased VSG levels in the knockdown cells, this effect is likely even more pronounced.

### 3.8 Effects of ERAP32 and ERAP18 RNAi on VSG exocytosis

To address whether ERAP32 or ERAP18 are required for VSG exocytosis, we performed a VSG export assay. In this assay, a shift from membrane-anchored VSG to soluble VSG in hypotonic lysates, which occurs with identical kinetics to arrival at the cell surface, is monitored via movement of [^35^S]-metabolically-labeled VSG (Bangs et al.,1986; Allen et al., 2003).

**Fig. 7:**
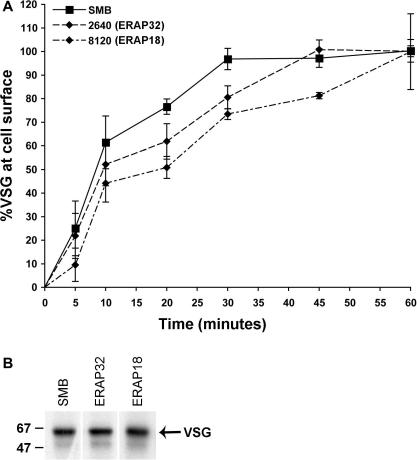
Effect of silencing ERAP32 or ERAP18 on VSG exocytosis. (A) VSG export was monitored by pulse-chase labeling with [^35^S]-labeled methionine/cysteine, followed by hypotonic lysis to release surface VSG via action of endogenous GPI-PLC. Data represent the kinetics of newly synthesized VSG transported from the endomembrane system to the cell surface. The experiment was performed as described in Allen et al., 2003. Data were from two independent experiments and error bars indicate the standard error. (B) Metabolic labeling of newly synthesized VSG. Newly synthesized VSG within 10 min were labeled with [^35^S]-labeled methionine/cysteine and detected by autoradiography. The experiment was done twice with identical results. Lanes presented are from the same gel, and manipulated solely for presentation purposes.

We monitored VSG export for up to 60 min, taking samples from parental SMB cells, and ERAP32 and ERAP18 RNAi cells. The kinetics of VSG export for all three cell lines was similar (Fig. 7A). However, a detectable delay in VSG export for both RNAi cell lines was found. These data suggest that while there is clearly no block to VSG export and delivery to the cell surface, there is a decrease in the rapidity with which VSG molecules arrive at the surface. However, given the comparatively minor magnitude of this defect and the absence of clear accumulation of VSG within the cell, we conclude that exocytosis of VSG is, for the most part, functional and normal.

To check that overall synthesis of VSG was unaffected by RNAi of ERAP18 or ERAP32, we pulse-labeled cells for 10 min with [^35^S]-methionine and analyzed the resulting lysates by SDS–PAGE and autoradiography. These data demonstrated that VSG is synthesized in the knockdown cells at similar levels to SMB cells (Fig. 7B). The absence of increased VSG synthesis as a result of knockdown of ERAP32 and ERAP18 confirms that the augmented levels of bulk VSG detected must be due to slower degradation, and not increased synthesis.

### 3.9 ERAP32 and ERAP18 knockdown leads to morphological abnormalities

Based on the observations above, ERAP32 and ERAP18 clearly play a role in the expression of VSG, and also in maintaining normal ER morphology (Fig. 4). To investigate structural changes in more detail we analyzed cells induced for RNAi for 1 day for ERAP32 and ERAP18 by transmission EM. Compared to uninduced cells, induced cells contain extensive vesicularization and disorganized ER, as well as prominent autophagosomes/multivesicular bodies (MVBs).

**Fig. 8:**
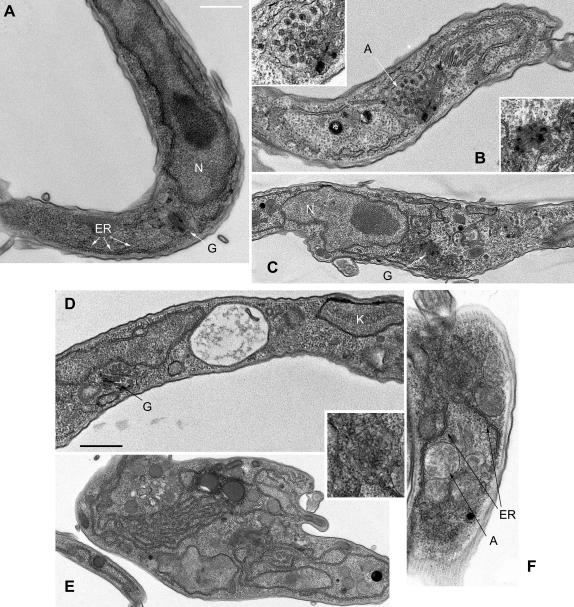
Transmission electron micrographs of ERAP32 and ERAP18 RNAi cell lines. (A) Uninduced ERAP32 RNAi cells. (B and C) Induced (24 h) ERAP32 RNAi cells. Top inset; MVB-like structure from panel B. Lower inset; golgi apparatus from panel C. (D) Uninduced ERAP18 RNAi cells. (E and F) Induced (24 h) ERAP18 RNAi cells. Inset; enlargement of vesicular globular cluster from panel F. N; nucleus, K; kinetoplast ER; endoplasmic reticulum, G; Golgi apparatus, A; autophagosome or multivesicular body. Scale bars: 500 nm.

For ERAP32, there was evidence of ER hyperplasia, and the appearance of large autophagosomes, which likely indicate the activation of an ER stress response. The Golgi complex did appear in many cells to retain normal morphology (Fig. 8A, C and D), but the appearance of fragmented and disorganized membrane structures associated with the ER was also prominent (Fig. 8B and C). The phenotype for ERAP18 was more pronounced, with, in some cases, extreme ER hyperplasia (Fig. 8E) and the appearance of very prominent vesicle clusters, possibly of ER or Golgi complex origin (Fig. 8F inset), together with autophagosomes/MVBs. Importantly these morphological changes appeared restricted to the ER and associated elements, and other structures appeared relatively unaltered. Induction of autophagosomes is a common response to ER-related stresses, consistent with changes to ER function and structure. The emergence of the changes also confirms both the light microscopy data and that ERAP32 and ERAP18 are apparently required for the correct morphology of the ER.

## 4 Discussion

Despite an obvious major impact on host-parasite interactions, and hence virulence, comparatively little is known concerning construction and maintenance of protozoan parasite surfaces. For *T. brucei* most focus has been on the dominant surface antigen, VSG, due to both its prominent role in antigenic variation and as a model GPI-anchored protein. However, there remain many additional proteins on the trypanosome cell surface that perform multiple functions, including nutrient uptake via receptors or transporters (transferrin, haem, nucleosides and nucleotides (reviewed in Field and Carrington 2009; Bridges et al., 2008)), putative signaling functions (adenylate cyclases (Seebeck et al.,2004)) and the high abundance ISGs, whose functions remain cryptic (Field and Carrington, 2009). Critically, maintenance of both protein quality and copy number requires monitoring folding within the exocytic system and balance between biosynthesis, ER-associated degradation and turnover from the surface pool. Identification of specific factors involved in maintaining VSG and other surface antigen copy numbers, folding quality and export efficiency remains poorly explored. In a related study we analyzed a cohort of conserved chaperones and folding factors using RNAi, with the major findings that VSG is made in two to threefold excess, has a considerable chaperone requirement for folding and is subject to an efficient ERAD/quality control mechanism (Field et al., 2010). Specifically, several heat-shock family proteins, the calreticulin/glucosyltransferase monitoring system, together with thiol oxidoreductases of the disulfide isomerase (PDI) family, are all implicated in VSG ER quality control. A common phenotype is decreased proliferation, abnormal ER morphology and increased levels of VSG in total cell lysates. Additionally, in some cases we also detect increased ISG levels.

In this study we took a new approach, and specifically searched the kinetoplastid genome databases for candidate trypanosome-specific ER-localized factors that may play a role in controlling the surface proteome. Of four candidate proteins that are restricted to the kinetoplastida and bear degenerate C-terminal ER-retention signals, we were able to demonstrate an ER location for two, which we designated ERAP18 and ERAP32; both have clearly profound effects on bloodstream stage proliferation in *in vitro* culture. The remaining two gene products, Tb927.7.3870 and Tb927.2.5140 had a less profound impact in cell proliferation, which may reflect a less important role or inefficient knockdown and, as we were unable to confirm their ER location despite multiple strategies attempting to tag the ORF or overexpress a tagged fusion protein, they were not investigated further.

Knockdown of ERAP18 and ERAP32 also revealed a role in VSG metabolism, as a specific increase in VSG in whole cell lysates was detected in cells depleted of these gene products, and suggesting a possible role in ERQC. Surprisingly there was no obvious increase to internal pools of VSG, as detected by surface biotinylation or immunofluorescence, ruling out an exocytic block, the latter confirmed by direct analysis of VSG export kinetics. Differentiating the phenotype from the chaperones studied elsewhere was the absence of an effect on ISG65 or ISG75 protein levels. Further, increased levels of VSG were not due to augmented biosynthesis or an increase in the cell surface area. Finally, ultrastructural analysis suggests that both ERAP18 and ERAP32 are required for maintaining normal ER structure.

Based on their locations and the morphological changes obtained following knockdown, we propose that ERAP18 and ERAP32 function at the ER. Therefore, the alterations to VSG copy number and VSG density at the cell surface are a consequence of depletion of ERAP18 and ERAP32 from the ER. As we do not observe intracellular accumulation of this excess VSG, the presence of excess VSG at the cell surface rules out a simple role in ERQC for these two gene products, and further suggests that the excess VSG is correctly folded. The absence of orthologues in higher eukaryotes, or a common domain, also occludes any facile prediction of function. However, there are some clear differences in the anterograde transport pathway requirements for GPI-anchored proteins between species; for example a requirement for sphingolipid/sterol synthesis in *S. cerevisiae* is not observed in trypanosomes (Horvath et al., 1994; Sutterwala et al., 2007). The increase in surface VSG density suggests the presence of a counting mechanism for ER export of VSG in normal cells, and that this mechanism has been disrupted by knockdown of ERAP18 and ERAP32, implicating these gene products as part of that mechanism.

In higher eukaryotes several anterograde signals are important for controlling the efficiency of incorporation of export cargo into transport vesicles at the transitional ER (Nishimura and Balch 1997). There is good evidence for both selective export of GPI and *trans*-membrane domain anchored proteins in higher eukaryotes, as well as for a role for p24 cargo receptors in facilitating incorporation of cargo into transport vesicles (Mayor and Riezman, 2004; Fiedler et al., 1996). More recently a role for 14–3-3 proteins in anterograde transport has been uncovered, whereby the 14–3-3 protein recognizes the diarginine motif within the cytoplasmic domain of *trans*-membrane domain cargo, and competes for binding with COP I subunits (Yuan et al., 2003). This mechanism likely plays a role in both ERQC, monitoring complex assembly as well as retarding export of some proteins, for example MHC Class I (Boyle et al., 2006). Many of these factors are conserved in trypanosomes, for example orthologues of p24 and 14–3-3 proteins are encoded in the genome, while both COP I and COP II coats are fully represented. Recent evidence indicates that VSG exits the ER via a conventional COP II-dependent route and depends on the functions of specific Sec23/24 paralogues (Sevova and Bangs 2009). However, the transitional ER/ER exit sites (ERES) may be spatially restricted in trypanosomes, and coordinated with the Golgi complex (Sevova and Bangs 2009).

Morphological distortion of the ER following ERAP18 and ERAP32 knockdown certainly suggests disruption of close coupling between the ERES and Golgi apparatus, and likely explains the slight delay in VSG export kinetics. Further, if a selection or counting mechanism for VSG is present at the ERES, then this likely was also disrupted in the knockdown cells and may permit excess VSG to escape from the ER and into the Golgi complex, and potentially the surface. However, the localization of ERAP18 and ERAP32 suggests that they are not specifically localized at the ERES, and therefore their functions are unlikely to be ERES-specific. Significantly, while there is heterogeneity in the ERES proteome in *S. cerevisiae*, indicative of differentiated function (Castillon et al., 2009), it is unknown if such diversity is present in trypanosomes. The requirement for specific Sec23/24 paralogues for VSG export may reflect some degree of functional diversification in ER exit sites or COP II coats (Sevova and Bangs 2009). The contributions of ERAP18 and ERAP32 to this potential mechanism are unknown, but may in part explain the lack of effects of the knockdowns on ISG65 or ISG75 expression levels.

Finally, most startling is the observation that the trypanosome is able to accommodate additional VSG into the surface coat. While this is inconsistent with the concept of a saturated surface (Mehlert et al., 2002), it is compatible with the ability of the parasite to rapidly cap and turn over VSG (Engstler et al., 2007; Pal et al., 2003) and also for recognition of ISG epitopes at the cell surface of living cells (Chung et al., 2004). The basic architecture of the African trypanosome cell surface, a potent device for defense against the mammalian immune system, remains enigmatic and likely holds more secrets that remain to be uncovered.
